# MTSTR: Multi-task learning for low-resolution scene text recognition via dual attention mechanism and its application in logistics industry

**DOI:** 10.1371/journal.pone.0294943

**Published:** 2023-12-12

**Authors:** Herui Heng, Si Li, Peiji Li, Qianfeng Lin, Yufen Chen, Lei Zhang

**Affiliations:** 1 Institute of Logistics Science and Engineering, Shanghai Maritime University, Shanghai, China; 2 Institute of Logistics, Yunda Express Co., Ltd, Shanghai, China; 3 Department of Computer Engineering, Korea Maritime and Ocean University, Busan, South Korea; 4 School of Automation, Northwestern Polytechnical University, Shanxi, China; National University of Sciences and Technology NUST, PAKISTAN

## Abstract

Recognizing texts in images plays an important role in many applications, such as industrial intelligence, robot vision, automatic driving, command assistance, and scene understanding. Although great progress has been achieved in various fields, research on complex systems modeling using text recognition technology requires further attention. To address this, we propose a new end-to-end multi-task learning method, which includes a super-resolution branch (SRB) and a recognition branch. To effectively learn the semantic information of images, we utilize the feature pyramid network (FPN) to fuse high- and low-level semantic information. The feature map generated by FPN is then delivered separately to the super-resolution branch and the recognition branch. We introduce a novel super-resolution branch, the SRB based on the proposed dual attention mechanism (DAM), designed to enhance the capability of learning low-resolution text features. The DAM incorporates the residual channel attention to enhance channel dependencies and the character attention module to focus on context information. For the recognition branch, the feature map generated by FPN is fed into an RNN sequence module, and an attention-based decoder is constructed to predict the results. To address the issue of low-resolution text recognition in numerous Chinese scenes, we propose the Chinese super-resolution datasets instead of relying on traditional down-sampling techniques to generate training datasets. Experiments demonstrate that the proposed method performs robustly on low-resolution text images and achieves competitive results on benchmark datasets.

## Introduction

**Fig 1:**
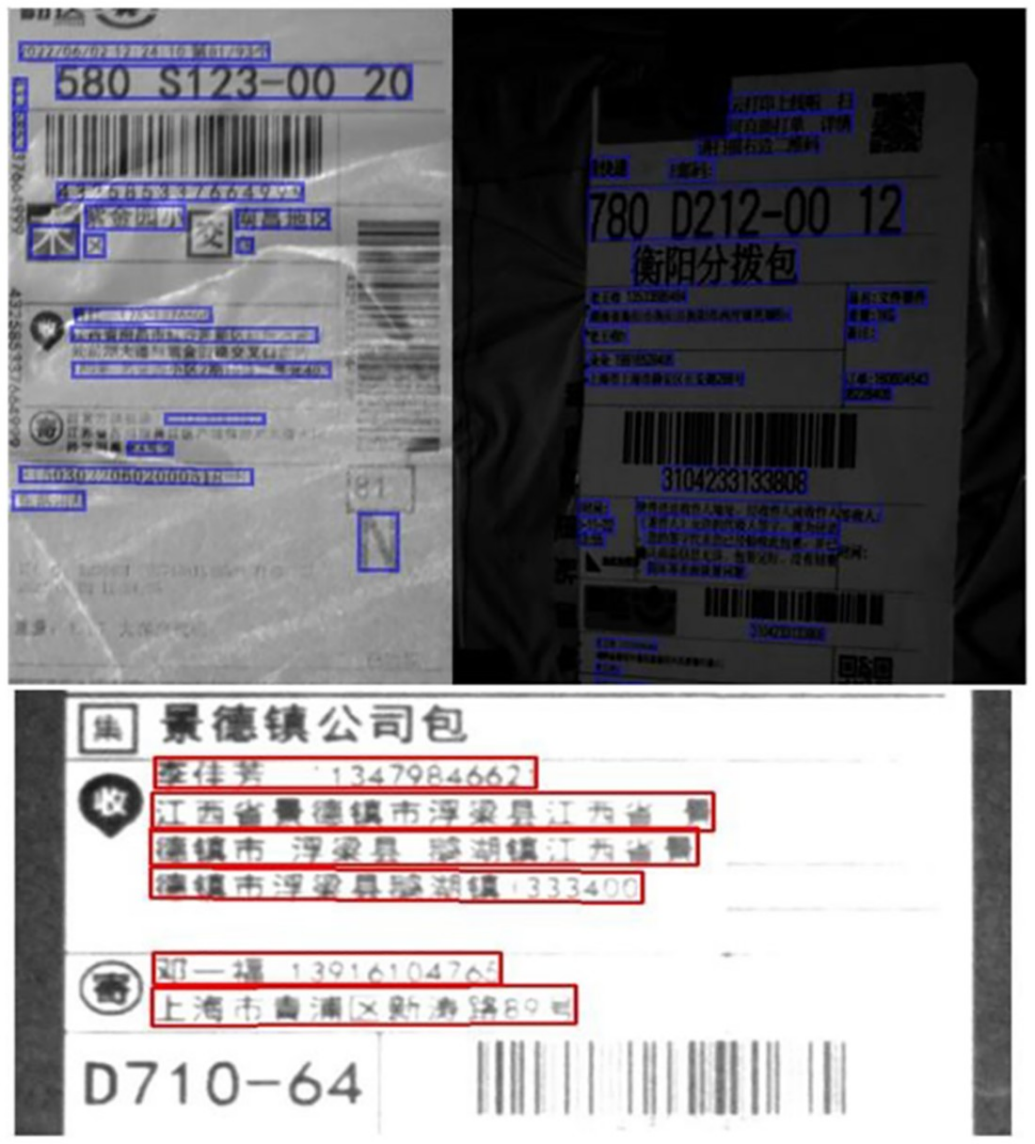
The cropped delivery sheet images were in low-resolution condition. We have detected the key information in blue box or red box by text detector.

Complex systems have garnered significant attention, and effectively addressing complex system applications through information theory modeling can greatly enhance industrial intelligence. Logistics information is widely acknowledged as a pivotal factor in parcel transportation and essential for customer communication. China’s e-commerce industry has witnessed substantial growth over the past decade, driven by advancements in Internet technology, which has in turn fueled the expansion of the logistics sector. With the nation generating more than 100 million express parcels daily to meet domestic demand, the logistics industry has become a cornerstone of economic development. However, efficiently managing this immense daily parcel volume presents a significant challenge. Beyond automation technologies, Scene Text Recognition (STR) technology has emerged as a crucial solution. STR not only facilitates intelligent sorting in distribution centers but also enables rapid extraction of customer information during the final stage of distribution. By replacing labor-intensive manual processes, STR offers substantial cost savings for businesses contending with rising labor expenses. In distribution centers, STR technology captures destination codes from express parcels, enabling intelligent routing to centralized areas before final delivery. Although text recognition research has been applied in various domains, such as bank slip recognition, shopping receipt recognition, and passport recognition, limited attention has been directed toward the logistics field. This is because delivery sheet images captured by cameras are often bent, distorted, and low in resolution, as exemplified in Fig 1. As is shown in Fig 1, the text in express sheet image is extremely in low-resolution condition and some Chinese characters cannot be recognized because of the low-resolution captured by the camera.

Great progress has been made in scene text recognition, but the accuracy of low-resolution text recognition has not reached a satisfactory level due to various influencing factors. Many blurred and low-resolution images exist in natural scenes, and these pose serious challenges to text recognition.

The progress in scene text recognition (STR) has significantly benefited from the remarkable performance of Convolutional Neural Networks (CNN) and Recurrent Neural Networks (RNN). Generally, an STR model consists of four main components: rectification, feature extraction, sequence module, and decoder module. Prior research [1–4] has introduced enhanced models for recognizing curved text images, employing a rectification module to rectify text images and recognition capabilities. Furthermore, many STR approaches incorporate a transformer module or a gated recurrent convolution network [5] to address challenges, especially for curved text. Thin-plate splines (TPS) [6, 7], a variation of Spatial Transformer Networks (STN) [8], exhibit effective rectification performance. Reference [9] conducted a study comparing the performance of VGG [10], RCNN [3], and ResNet [11] as feature extraction modules, revealing that deep models can achieve superior results in STR. Concerning the sequence module, references [7, 12] applied bidirectional Long Short-Term Memory (BiLSTM) to enhance sequence modeling. Baek et al. [13] extended this comparison by adding or removing the BiLSTM module in their proposed method, illustrating that the inclusion of BiLSTM enhances text sequence recognition. Finally, the decoder module utilizes Connectionist Temporal Classification [12] as the decoding mechanism to predict the character sequence.

Recent studies [14–16] have made progress in text recognition performance on various benchmark datasets, but they have not explored the domain of low-resolution text recognition. Previous single image super-resolution approaches [17–20] were developed to generate high-resolution features; however, they were trained on low-resolution datasets created through simple down-sampling, which is unsuitable for real low-resolution text recognition. Furthermore, logistics delivery sheets containing numerous low-resolution Chinese characters, exhibit various situations including blurring, missing strokes, sticking strokes, and motion blur. To address these challenges, we propose the Chinese Text Super-Resolution Dataset (CTSD), a paired dataset consisting of low-resolution and high-resolution samples specifically designed for the super-resolution task. Our literature review indicates that there have been no previously proposed Chinese paired super-resolution datasets, which we introduce in Chinese super-resolution dataset explanation section.

In our study, we present a novel text recognition method and introduce an efficient super-resolution branch aimed at continuous improvement of recognition results. Our key contributions are as follows: (1) We create a new Chinese super-resolution dataset that includes commonly used Chinese characters, numbers, and English letters. The dataset comprises Chinese articles and desensitized Chinese address texts. (2) We propose a multi-task learning approach for scene text recognition, which includes a text recognition branch and a super-resolution branch. The proposed super-resolution branch, incorporating residual super-resolution units, effectively captures rich information from low-resolution features. It is utilized to generate super-resolution text features by contrastive learning between high-resolution and low-resolution features. (3) We design a dual attention mechanism as a significant component of our super-resolution branch (SRB). We introduce the residual channel attention (RCA) to comprehensively capture channel-wise dependencies and propose a character attention module to capture contextual information between pixels while preserving essential information for global understanding. (4) To model the inter-character relationships within the text sequence, we construct an attention-based decoder. Experimental results on low-resolution benchmark datasets demonstrate the superiority of our proposed method over recent efficient text recognizers, establishing it as an effective solution for recognizing low-resolution text images in complex logistics scenes.

## Related work

From a problem-solving perspective, previous related works have focused on two directions, one is the text recognition for curved text images in natural scenes, and the other is text recognition for low-resolution text images.

### Text recognition methods for curved text images

Traditional optical character text recognizers cannot meet the complex recognition requirements, dynamically changing text scenes have spurred the development of numerous STR methods. Researchers have turned to deep learning models to devise innovative solutions. In an effort to enhance the recognition of curved text images, Shi et al. [7] introduced an automatic rectification module to combat curved text. They proposed a spatial transformation network to convert images into a horizontal and more legible format, effectively rectifying various forms of curved text and improving curved text recognition. To streamline the complexity of the rectification module, Shi et al. [15] introduced a novel rectification network. They implemented a thin-plate spline transformer integrated with an attention mechanism, enabling direct control over curved text points and straightening them into a horizontal orientation. Distinguished from [7, 15], Zhan et al. [21] presented a repetitive rectification framework capable of continuous text region rectification, leading to enhanced text recognition performance. In contrast, references [7, 15] rectified text regions only once. Another significant advancement came from Lee et al. [22], who proposed adaptive 2D positional encoding to bolster feature extraction and address curved images. Their approach yielded remarkable results on public datasets, particularly excelling on curved datasets thanks to the inclusion of self-attention layers in its encoding component, which proved highly beneficial for curved text images. Despite these remarkable contributions to curved text recognition, none of these methods have effectively tackled the challenges posed by low-resolution text images.

### Text recognition methods for low-resolution images

To tackle the challenge of low-resolution text images, especially given the limited use of semantic information in most current models for text recognition, researchers have proposed various innovative approaches. Yu et al. [23] introduced a novel Semantic Reasoning Network (SRN) to capture rich semantic context information. SRN effectively combines visual and semantic context information, segmenting characters individually and then aligning them horizontally. This method demonstrated improved performance on publicly available datasets. Inspired by the effectiveness of language models in supervising word order generation, Qiao et al. [24] incorporated a pre-trained language model to supervise encoding and decoding processes. Predictive semantic information training was supervised by a pre-trained word embedding model, making this approach more suitable for addressing low-resolution text recognition. Zhang et al. [25] adopted a text recognition framework with automatic search capabilities. This adaptable framework can be fine-tuned for different datasets, drawing inspiration from neural framework search. For recognizing unsupervised sequence data, Zhang et al. [14] proposed a sequence-to-sequence control adaptive network. They employed gate attention similarity units to adjust attentional information distribution, enhancing feature information. To augment the semantic feature, global context block [26] was introduced into text recognition. In contrast to RNN-based decoder [27], Li et al. [28] presented a transformer-based method, leveraging the self-attention mechanism for Optical Character Recognition (OCR). This approach proved highly effective in addressing low-resolution text images, thanks to the self-attention layers’ exceptional information-carrying capabilities. Wan et al. [29] developed an analytical framework tailored for low-quality images. Their semantic-based segmentation model adeptly utilizes visual features to generate improved results.

However, these two kinds of methods do not explore the effectiveness of the super-resolution network for STR, which is what we wish to focus on in this study. Comparing these methods, we pay attention to the use of the efficient super-resolution module for solving low-resolution images.

## Introduction of Chinese super-resolution dataset

### Chinese super-resolution dataset annotation

Considering the insufficient availability of Chinese datasets for real scene text recognition, we are aware that while there are 26 English letters, there are more than 5000 commonly used Chinese characters in Chinese scenes. This presents significant challenges for Chinese scene text recognition. Additionally, it is crucial that the training images closely resemble real scene images, including blurred, missing-stroke, and motion-blurred images, to meet the requirements. Traditional single image super-resolution approaches were trained on datasets generated through down-sampling. However, down-sampling is a simplistic and inappropriate method for real low-resolution text recognition. To tackle these challenges, we have developed a novel Chinese super-resolution dataset specifically designed for Chinese scene text recognition.

The proposed Chinese Text Super-Resolution Dataset (CTSD) includes numbers, English letters, and more than 5000 commonly used Chinese characters. The corpus consists of Chinese articles and desensitized Chinese address text, such as specific communities and streets. To create the super-resolution images, we first extract four to ten Chinese characters from the corpus and then paste them onto background images to generate high-resolution images. Subsequently, to generate corresponding low-resolution images, we employ five different methods to transform the high-resolution images into a low-resolution condition. In total, we have generated 5.67 million high-resolution images, resulting in 5.67 million low-resolution images after the transformation process. We randomly divided the 5.67 million low-resolution images into two parts: 70,000 images for the fixed testing sets and the remaining images for the fixed training sets, as shown in Table 1.

**Table 1:** Statistics of the proposed Chinese Text Super-resolution Dataset (CTSD).

Chinese Super-resolution Dataset	Number of LR images	Number of SR images
Number of training sets	5.6 million	5.6 million
Number of testing sets	70 thousand	-

### Chinese super-resolution dataset explanation

First, we produced 5.67 million high-resolution datasets and then processed them in a low-resolution condition by designing the processing functions, such as blur, stroke sticking, left-right motion blur, up-down motion blur, and missing stroke. We use the ‘random.randint()’ function from the Python language to generate numbers from 0 to 9. It returns a random integer. The random.randint (a, b) function uses a random seed and an algorithm to generate a random number, which is then mapped to the range [a, b] through mathematical operations. This ensures that the generated integers are uniformly distributed and within the specified range, hence, it can generate a variety of low-resolution text images with a uniform distribution by selecting the function corresponding to the generated random p. The principle is as follows:

$$\begin{array}{c} \left\{ \begin{array}{cc} F_{s-s} & p=9\\ F_{s-d} & p=8\\ F_{\text{udmb}\;} & p=7\\ F_{\text{rlmb}\;} & p=6\\ F_{\text{gau}\;} & p=5\\ I & p\in [0,4] \end{array}\right. \end{array}$$ (1)

where each p can determine one processing function, *I* denotes that if *p* ∈ [0, 4], the generated text images will not be processed. *F*_gau_ denotes the blur function, *F*_rlmb_ denotes the right-and-left motion blur function, *F*_udmb_ denotes the up-and-down motion blur function, *F*_*s*−*d*_ is the missing stroke function (the stroke is missing a part of it), and *F*_*s*−*s*_ is the stroke sticking function indicating that the stroke appears to be in bold.

The motion blur of the image is expressed mathematically as follows:

$$\begin{array}{c} dst\left(x,y\right)=\sum_{\begin{array}{c} 0<x^{\prime }<kernel.cols\\ 0<y^{\prime }<kernel.rows \end{array}}kernel\left(x^{\prime },y^{\prime }\right)*src\left(\left(x+x^{\prime }-anchor.x\right),\left(y+y^{\prime }-anchor.y\right)\right) \end{array}$$ (2)

where *dst*(*x*, *y*) is the processed motion blur image, src(*x*, *y*) denotes the original images, and (*x*, *y*) is the image pixel value.

### Details of processing functions

#### Blurred images

**Fig 2:**
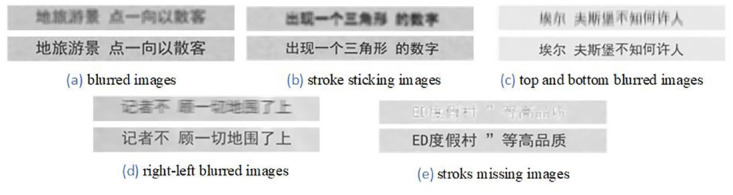
Examples of our proposed super-resolution Chinese dataset with paired low and high-resolution images. The upper image is low-resolution image which made by the corresponding function, the image which is in below is high-resolution image.

Blurred images exist widely in real scenes, and many characters are difficult to identify. We use the different kernels to blur the images randomly, and an example is shown in Fig 2(a). We use gaussian blur to generate blurred images. Larger kernel size results in more significant image blurring. In our experiments, we first read the high-resolution images, and apply each kernel to the original image to generate blurred images, and then calculate the RMSE (Root Mean Square Error) between each blurred image and the original image. In this study, we set the kernel size from 3 to 15, and the kernel size is the odd. If the RMSE is greater than the threshold, it indicates that the image is too blurred and cannot be recognized. Too blurred images will cause the model to focus too much on low-level feature information, which will have a negative impact on the recognition branch. In the process of creating our Chinese super-resolution dataset, we found that when the RMSE is greater than 6.5, text images become excessively blurred and difficult to distinguish. Therefore, we set the threshold to 6.5, ensuring that the RMSE of blurred images is less than 6.5.

#### Stroke sticking

Stroke sticking is handled by using image morphology processing methods, and an example is shown in Fig 2(b). First, we use the cv2.getStructuringElement function from OpenCV to create a matrix that defines the shape and size. Then, we apply the created matrix to the input image using the erode function. The erosion operation moves the matrix across the image, it retains the minimum pixel value in the covered area where the matrix shape overlaps with the image. This results in a gradual reduction or elimination of white areas in the image, achieving a stroke sticking effect.

#### Up-and-down motion blur

Up-and-down blur is operated by processing the middle row of the kernel numerically as a new convolutional kernel to simulate the up-and-down motion of the images, an example is shown in Fig 2(c). We use the filter2D function from OpenCV to achieve up-and-down directional blurring of images. We set the kernel size from 5 to 11. For each kernel, we perform the following operation: the middle row of the kernel contains positive values, while the values in the other rows are set to 0. We also calculate the Root Mean Square Error (RMSE), and when it exceeds the threshold of 6.5, we increase the kernel values and recalculate.

#### Right-and-left motion blur

An image is motion blurred when a large number of images in real scenes are dynamically captured by cameras. The motion blur function makes images blurred in the right-left direction, and these images are processed by operating the middle column of the kernel numerically as a new convolution kernel. An example is shown in Fig 2(d). We use the filter2D function from OpenCV to achieve left-right directional blurring of images. We set the kernel size from 5 and 11. For each kernel, we perform the following operation: the middle column of the kernel contains positive values, while the values in the other columns are set to 0. We also calculate the Root Mean Square Error (RMSE), and when it exceeds the threshold of 6.5, we increase the kernel values and recalculate.

#### Missing stroke

Missing stroke is the most challenging condition in real scenes. We simulate the missing stroke characters, which can be processed by morphology algorithms, such as dilation and erosion, to achieve missing stroke in Chinese characters. An example is shown in Fig 2(e). To achieve a missing stroke effect, we first convert the input image to a gray-scale image. Then, we apply a dilation operation to gradually expand the text strokes, followed by an erosion operation to gradually reduce the strokes, thereby simulating the missing effect. This can be accomplished using OpenCV’s cv2.dilate for dilation followed by cv2.erode for erosion. Adjusting parameters such as the size of the structural element and the number of iterations in the dilation and erosion operations allows control over the intensity of the missing stroke effect.

## Proposed approach

### Overall framework

**Fig 3:**
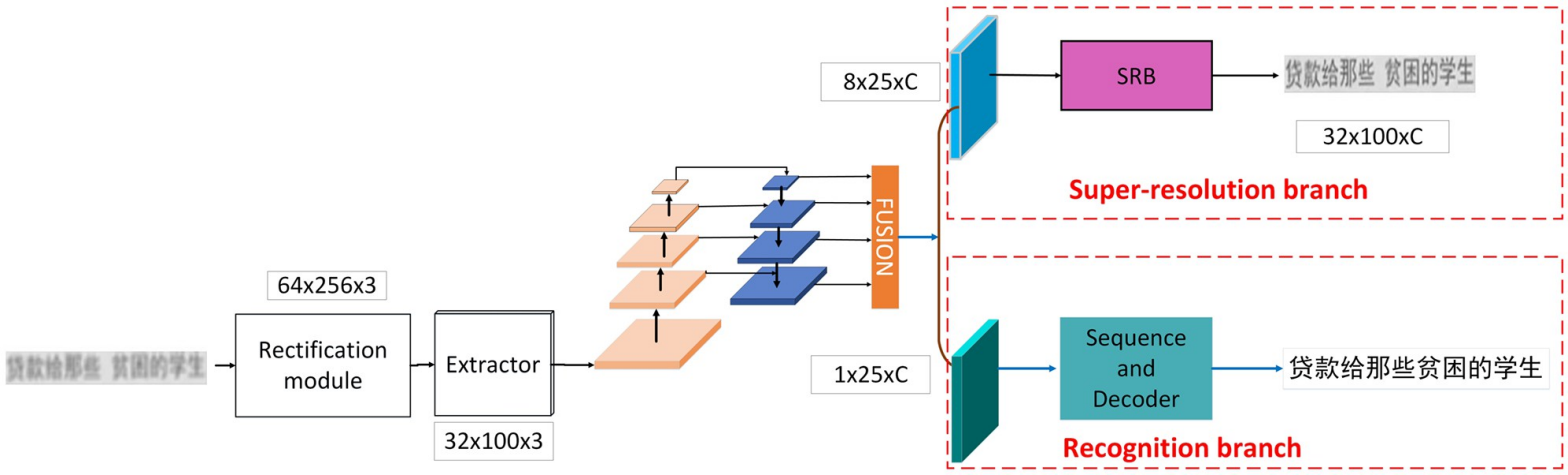
Overall framework of our proposed method.

The framework of proposed model is shown in Fig 3. Our proposed model includes the rectification module, the feature extraction module, FPN, the text recognition branch and super-resolution branch. Text recognition branch contains a sequence module, and a decoding module. Super-resolution branch contains the super-resolution units, each unit includes the dual attention module (DAM) and convolution layers. We design a super-resolution branch (SRB) so that the network can continuously learn the difference between low- and high-resolution features, the capability of text recognition will be improved by enhancing the features resolution. To model the inter-character relationships of the text sequence, we constructed an attention-based decoder in recognition branch.

### Rectification module

The framework of rectification network is based on [7], which includes localization module, grid generator and sample generation. The localization module is used to detect the control points in the graph and output the location of the control points. The grid generator calculates the mapping relationship of each point corresponding to the control points and generates the coordinate position of the points {*P*_1_⋅⋅⋅*P*_*n*_}. The sampling generator samples the point positions to generate a rectified graph. The text is firstly rectified into a horizontal layout before feature extraction, which exhibits good performance for non-horizontal layout of text images.

### Feature extraction and feature pyramid network

We used Resnet34 [11] to extract text image features. The shape of the last layer of the extraction module is $\frac{W}{4}\times \frac{H}{32}\times C$, where W denotes the width of the input image and H denotes the height of the input image. After fusing the high- and low-level semantic information by the FPN network, the fused feature map is adopted as the input of the recognition branch and super-resolution branch, respectively.

### SRB

**Fig 4:**
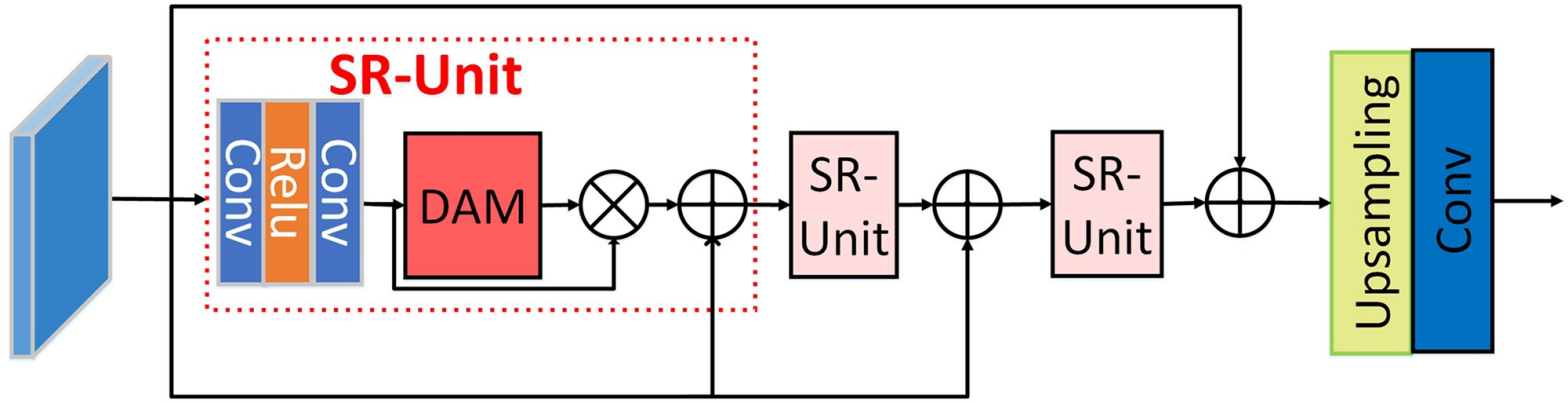
Architecture of our proposed super-resolution branch (SRB). Each SR-unit includes the convolution layers and the proposed dual attention module (DAM). One of SR-UNIT is shown in red dotted box.

We designed super-resolution branch so that the network can continuously learn the difference between low- and high-resolution features in backward learning, which is shown in Fig 4. SRB contains the designed residual super-resolution units which can grasp abundant information from low-resolution features. Each super-resolution unit includes a convolution block and the dual attention network mechanism (DAM).

**Fig 5:**
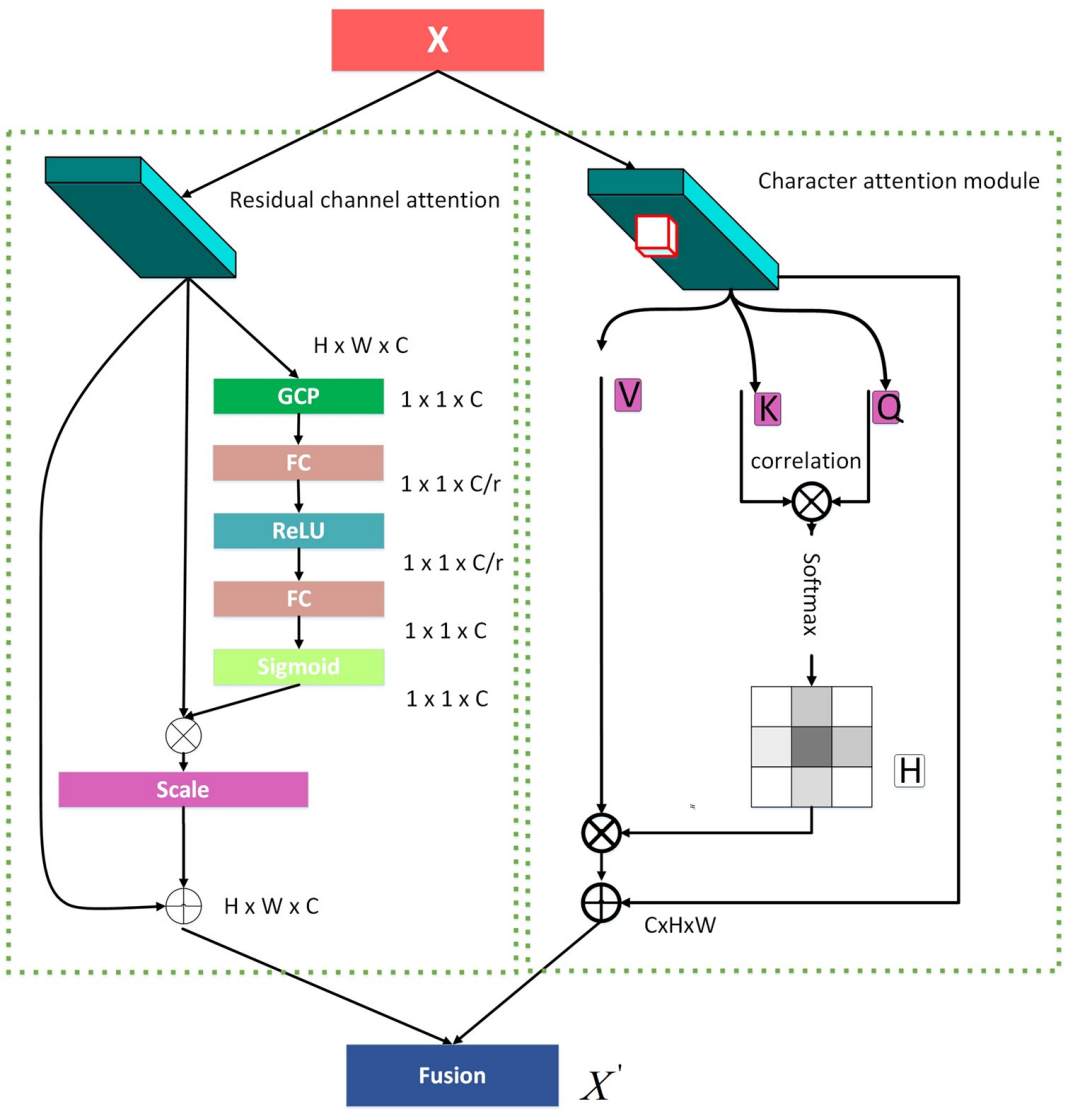
DAM module. The left part is the residual channel attention, the right part is the character attention module.

We will describe the dual attention mechanism as follows, as is shown in Fig 5, it contains the residual channel attention (RCA) and the character attention module (CAM).

#### RCA

We design the residual channel attention (RCA) as the significant part of our DAM, which aims to fully capture channel-wise dependencies. The structure of the RCA is shown in left part of Fig 5. We here briefly introduce the processing of RCA.

We get the feature map X, Let *X* = [*X*_1_, …, *X*_*C*_], the channel-wise statistics *h* ∈ *R*^*C*×1^ can be acquired by global covariance pooling. The C-th dimension of *h* is computed as:

$$\begin{array}{c} h_{c}=H_{GCP}\left(x_{c}\right) \end{array}$$ (3)

where *H*_*gcp*_ represents the global covariance pooling [30]. Compared with the commonly used first-order pooling, global covariance pooling explores feature distributions and captures feature statistics higher than the first-order pooling to obtain a highly discriminant representation.

To fully dig feature interdependencies from the aggregated information, a gating mechanism was applied as follows:

$$\begin{array}{c} w=f(W_{a}\delta (W_{b}\;h)) \end{array}$$ (4)

where *W*_*a*_ and *W*_*b*_ are the weight sets of convolution layer and used to set the channel size of the feature to C/r and C, respectively. f(⋅) and *δ*(⋅) denote the function of sigmoid and RELU.

Then, we calculate the output *μ* of RCA as follows:

$$\begin{array}{c} \mu =w\cdot X⊕X \end{array}$$ (5)

where X denotes the input of RCA and *w* is obtained by Eq (4), ⊕ denotes the element-wise addition.

#### CAM

As text in an image is organized as a sequence, it’s crucial to consider the adjacent row or column of a character when computing correlations. This is particularly important for text sequences that are curved, as it allows us to capture contextual information effectively. To address this, we’ve introduced a Character Attention Module (CAM) within our DAM. The CAM’s structure presented in the right part of Fig 5 enriches the local features X with context information, broadening the view of the context and selectively aggregating the context based on the spatial attention map.

Firstly, we get the 2D feature X, *X* ∈ *R*^*C* × *W* × *H*^, the character attention module first applies two convolutional layers with 1 × 1 filters on X to generate two feature maps M and N, respectively, {*M*, *N*} ∈ *R*^*C*′ × *W* × *H*^, *C*′ is the channel of feature map. After obtaining the feature maps M and N, it further generates the attention map H by correlation operation, the definition of the correlation operation is as followed:

$$\begin{array}{c} \varphi_{i,j}=M_{j}\cdot N_{i,j}^{T} \end{array}$$ (6)

*j* represents each position of $M,M_{j}\in R^{C^{\prime }}$, and *N*_*j*_ was extracted from N which are adjacent context columns or adjacent context rows among position j, the *N*_*i*,*j*_ is the i-th element of *N*_*j*_, *i* ∈ [1, …, *n*(*H* + *W* − *n*)], *φ*_*i*,*j*_ ∈ *D* is the degree of correlation between *M*_*j*_ and *N*_*i*,*j*_, *D* ∈ *R*^*n*(*H* + *W* − *n*) × (*W* × *H*)^, then a softmax was applied on D on the channel dimension to compute the attention map H, n denotes the number of adjacent columns or rows, we set n to 3 in this study. Meanwhile, X generates the V by the convolutional layer with 1x1 filters for feature adaption, and *V*_*j*_ was extracted from V which are adjacent context columns or adjacent context rows among position j, the context information is enriched by aggregation operation, which is defined as follows:

$$\begin{array}{c} \gamma_{j}=\sum_{i=0}^{n(H+W-n)}H_{i,j}\cdot V_{i,j}+X_{j} \end{array}$$ (7)

where *γ*_*j*_ is the feature vector in *γ* at position *j*, *γ* ∈ *R*^*C* × *W* × *H*^, *H*_*i*,*j*_ is the attention map by applying softmax on correlation matrix D.

Hence, the output of DAM:

$$\begin{array}{c} X^{{}^{\prime }}=f\left(\gamma ,\mu \right) \end{array}$$ (8)

where f denotes the fusion function, *μ* and *γ* denote the output of RCA, CAM.

#### Loss function of SRB

In order to generate high-resolution text features, we designed *L*_*sr*_ for learning the difference between low- and high-resolution features in backward learning. We applied the *L*_1_ loss function for *L*_*sr*_ as follows:

$$\begin{array}{c} L_{\text{sr}}=\frac{1}{N}\sum_{i=1}^{N}{∥(SR(I_{LR}^{i})-I_{HR}^{i})∥}_{1} \end{array}$$ (9)

where *L*_*sr*_ denotes the super-resolution loss, SR(.) refers to super-resolution branch, *i* is the indexing of images in a batch, and *I*_*LR*_ and *I*_*HR*_ are the corresponding low-resolution and high-resolution image features, respectively. In our experiment, we performed low-resolution processing on the raw text dataset as the input of our method, then, the raw text dataset is regarded as the ground-truth.

Additionally, to address the potential issue of slow execution when using the L1 loss in the super-resolution branch, we employed multiple strategies. Firstly, selecting more efficient optimization algorithms, such as Adam or RMSProp, can accelerate the computation of the L1 loss. Additionally, optimizing the model architecture can reduce the computational burden, increasing batch sizes improved computational efficiency (e.g., setting batch size to 64 or more), and employing parallel computing can distribute the computational load. Lastly, adjusting the hyperparameters of the L1 loss can strike a balance between computational efficiency and performance to address potential speed issues. In this study, the hyperparameter for the L1 loss is set to 0.05, as larger weights would lead to a sparser model but increase computational complexity. These strategies are combined and adjusted based on specific circumstances and requirements to enhance the computational efficiency of the model.

### Recognition module

#### Sequence module

We use a two-layer BiLSTM containing 256 cells to grasp the long-range dependencies in sequence module. The output of the BiLSTM module is a feature sequence *h*, *h* = (*h*_1_, …, *h_L_*), *L* is the length of feature map.

#### Decoder

The decoder module converts feature sequence into character sequence. Reference [12] proves that the STR is a sequence problem and employs the CTC mechanism [31] for decoding, which offers a differentiable mechanism that is insensitive to horizontal characters during end-to-end training, but it cannot model the inter-character relationships of the output and relies on external character language models. Here, we constructed an attention-based decoder as follows:

Firstly, the attention score feature map *μ* is calculated as follows:

$$\begin{array}{c} \mu (s_{t-1},h_{i})=\sigma (W_{2}\text{tanh}(W_{1}[s_{t-1};h_{i}]+b_{1})+b_{2}) \end{array}$$ (10)

where *σ* is sigmoid function, *W*_1_, *W*_2_, *b*_1_, *b*_2_ are the trainable parameters. Then, the attention score feature map *μ* is normalized to obtain *α*_*t*,*j*_:

$$\begin{array}{c} \alpha_{t,i}=\frac{\text{exp}(\mu (s_{t-1},h_{i}))}{\sum_{i^{\prime }=1}^{n}\text{exp}(\mu (s_{t-1},h_{i^{\prime }}))} \end{array}$$ (11)

Based on the feature sequence *h*_*i*_ generated by sequence module, we obtain the current context feature *c*_*t*_:

$$\begin{array}{c} c_{t}=\sum_{i=1}^{n}\alpha_{t,i}h_{i} \end{array}$$ (12)

At last, we calculate probability of predicting result *y*_*t*_ as following Eq (13):

$$\begin{array}{c} p(y_{t})=\text{sof}\;\text{max}(w_{3}f(s_{t-1},y_{t-1},c_{t})+b_{3}) \end{array}$$ (13)

$$\begin{array}{c} y_{t}∼p(y_{t}) \end{array}$$ (14)

where f denotes the function of long short-term memory (LSTM), p is a probability vector after the function softmax at t time. *W*_3_, *b*_3_ are the trainable parameters. *S*_*t*_ is the hidden state. The decoder module transforms *h* feature sequence into a length of T sequence after T step, whose output is (y1⋅⋅⋅ yT).

#### Loss function of the recognition

We used the cross-entropy loss function as the objective function of the recognition:

$$\begin{array}{c} L_{rec}=-\frac{1}{MN}\sum_{i=1}^{M}\sum_{j=1}^{N}y_{i,j}\;\text{log}(s_{i,j}) \end{array}$$ (15)

where *i* refers to the index of the sample in a batch, *j* refers to the index of the number in the label, *y* denotes the ground truth label, and *s* denotes the recognition result.

### Training strategy and loss function

Generally, a super-resolution network requires paired low-resolution and high-resolution datasets to compute the loss function. However, public text datasets usually only contain one type of dataset. Therefore, we performed low-resolution processing on the raw text dataset as the low-resolution training dataset.

The original datasets were processed under low-resolution conditions using the following processing principles:

$$\begin{array}{c} \left\{ \begin{array}{cc} F_{s-d} & i\;\text{mod}\;2=1,i/2\in (4,7,\ldots 3n+4)\\ F_{m-b} & i\;\text{mod}\;2=1,i/2\in (3,6,\ldots 3n+3)\\ F_{d-u} & i\;\text{mod}\;2=1,i/2\in (2,5,\ldots 3n+2)\\ F_{gau} & i\;\text{mod}\;2=1,i/2\in (1,4,\ldots 3n+1)\\ I & i\;\text{mod}\;2=0 \end{array}\right. \end{array}$$ (16)

where *i* refers to the index order of the image in the training folder: if it is even, it will not be processed; if it is odd, one of the functions will be used for low-resolution processing. *F*_*s*−*d*_ represents the function responsible for introducing missing strokes in text, a common occurrence in real scenes that pose recognition challenges. To simulate missing stroke characters, we employ morphology algorithms like dilation and erosion. *F*_*m*−*b*_ corresponds to motion blur, often observed in images dynamically captured in real scenes. The motion blur function involves processing the middle row of the kernel numerically as a new convolutional kernel to replicate the up-and-down motion in images. *F*_*d*−*u*_ signifies dynamic down-sampling, an alternative to the commonly used fixed fourfold down-sampling method. *F*_gau_ denotes the blurring method, where different kernels are randomly applied to blur images, generating diverse types of blurred images relevant to real-world scenarios.

We designed the following loss function for the entire framework:

$$\begin{array}{c} L_{\text{total}\;}=L_{rec}+\lambda L_{sr} \end{array}$$ (17)

where *L*_*rec*_ and *L*_*sr*_ are the recognition and super-resolution loss functions, respectively, λ is the hyperparamer of *L*_*sr*_.

## Experimental results and ablation study

### Commonly used evaluation datasets

To verify the effectiveness and robustness of the proposed model, we analyzed its performance on the following six public text datasets for evaluation:

1. IIIT5K [32] includes 5000 images, among which 2000 are for training and 3000 are for testing. The text is collected from street view and digital images.
2. SVT [33] has 647 images for testing. Most of the images in SVT are processed into blurred and low-resolution condition.
3. IC13 [34], some of which images are inherited from IC03 [35]. Non-alphanumeric images are removed. It includes 1015 testing words.
4. IC15 [9] was created by Google Glasses and is the most complex dataset in recent years. Most of its images have various distortions and blurred conditions. It contains 2077 cropped words for evaluation. It is the most challenging dataset including large number of low-resolution images.
5. SVT-P [36] contains 645 cut-out images from Google Street View that are randomly scrambled and have different perspectives. It is also a challenging dataset whose most of images are in the low-resolution condition.
6. CUTE80 [37] has 288 images for evaluation. It concentrates on curved text recognition. Most of the images have complex backgrounds and perspective scrambling.

### Implementation details

Our method was trained on SynText [38], Synth90K [39] and SynthAdd [40] to compare its accuracy against other text recognizers. To ensure uniformity in our experiments, we set the batch size to 512. The images are resized to 64 × 256 and fed into the rectification module. We utilized the ADADELTA optimizer [41] for the recognition branch to optimize the minimum objective and ADAM for the super-resolution branch.

### Experiment and analysis

#### Comparisons on CTSD

To demonstrate the performance of our proposed model on CTSD, we compared it with Aster [15], SEED [24], and DAN [42]. All of these models were trained and tested on CTSD, and the results are presented in Table 2. Data-Aug and SRB in Table 2 denote the data augmentation function and our proposed super-resolution branch, respectively.

**Table 2:** Performance of several methods on our proposed CTSD.

Method	Data-Aug	SRB	Accuracy (%)
Aster	✓	×	79.3
SEED	✓	×	80.2
DAN	✓	×	82.1
Ours without SRB	✓	×	80.5
Ours	✓	✓	85.3

As shown in Table 2, the experiments revealed that adding the super-resolution module improved the recognition rate for Chinese low-resolution images. The comparison between Aster and our model showed that our proposed model produced better recognition results with a 6.0% improvement. Our model also performed better than DAN with a 3.2% improvement in CTSD as shown in Table 2. When we removed the SRB from our model, we can get the finding that our method got the worse performance, the accuracy decreased by 4.8%.

**Fig 6:**
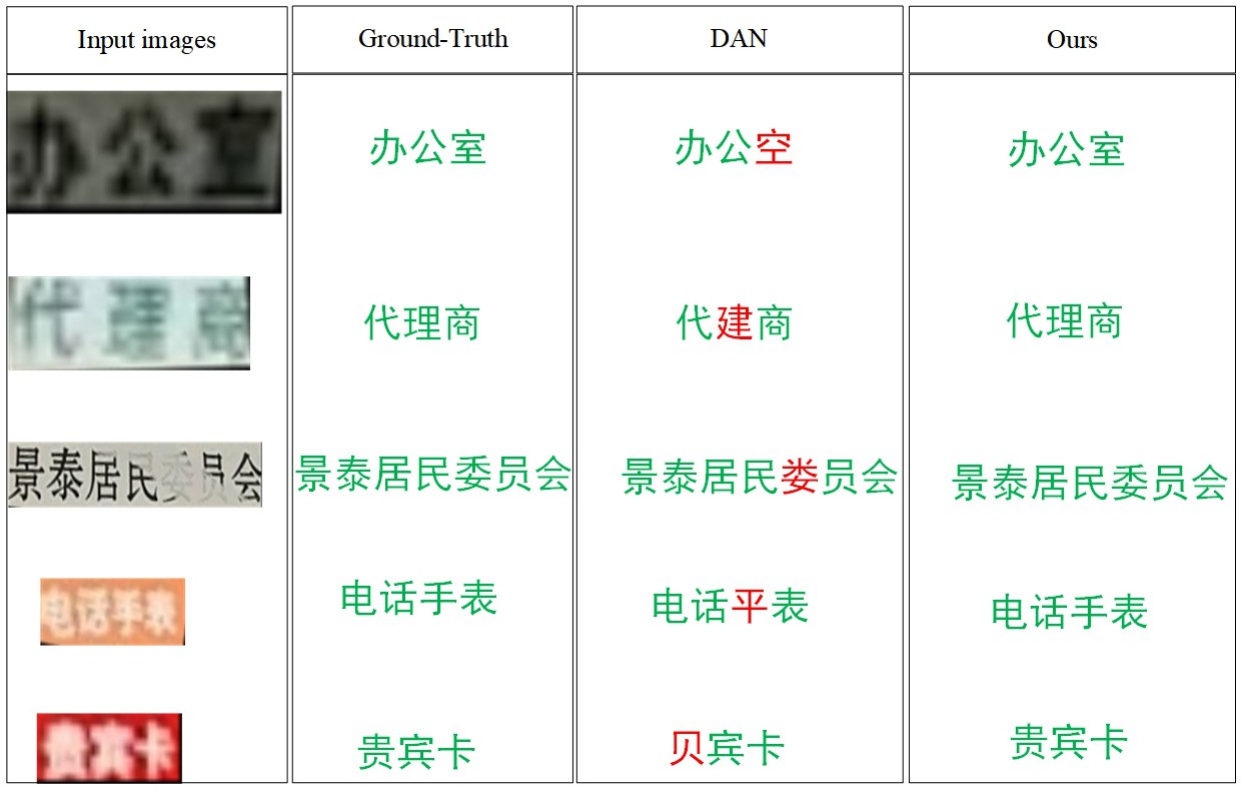
The comparison of models on real Chinese scene dataset.

We conducted a comparison between DAN and our proposed model, focusing on the recognition of challenging Chinese images through training on CTSD. To expedite convergence, we utilized DAN’s official website’s pre-training model. We tested several low-quality images from a real Chinese scene dataset provided by Baidu Company in China. As presented in Fig 6, our proposed method had a better performance than DAN in some low-resolution images. DAN wrongly recognized the characters in red font. This finding shows our proposed method with a super-resolution branch can learn better difference between low-resolution features and high-resolution features.

**Fig 7:**
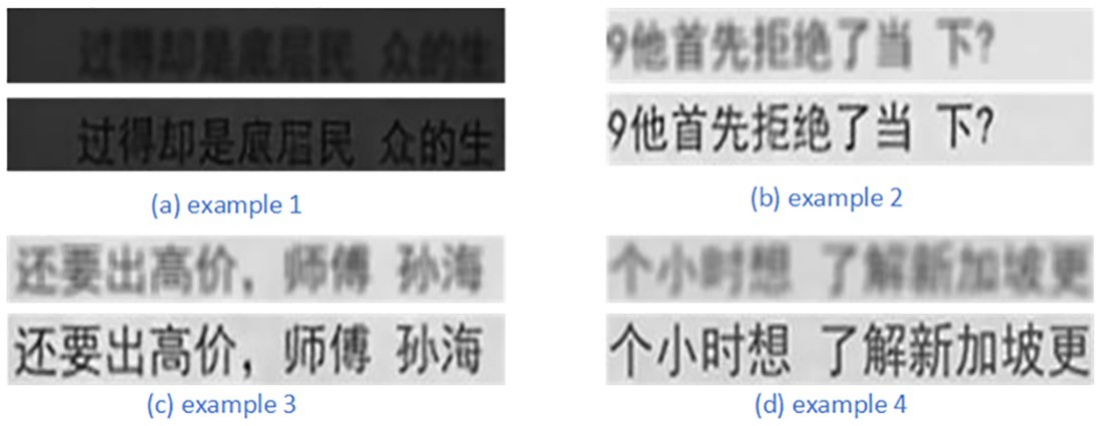
The effectiveness of super-resolution branch on Chinese low-resolution images.

We also selected some low-resolution data from CTSD to test the effectiveness of the super-resolution branch, and the results are presented in Fig 7. The below image generated by the super-resolution branch is more clear and higher resolution comparing the upper raw image in each example. This finding shows that SRB can effectively enhance low-resolution features.

#### Comparisons on public datasets and ablation study on SRB

To validate the effectiveness of our proposed super-resolution module, we compared our model with Aster [15], SEED [24], and DAN [42]. The training datasets used were SynText, Synth90K, and SynthAdd, while the testing datasets were SVT, IC13, IC15 and SVTP. IC15 consists of highly blurred images, while SVTP contains a significant number of low-resolution images. As demonstrated in Table 3, our model equipped with the SRB exhibited superior performance compared to these methods on SVT, SVTP, IC15, and IC13. Additionally, when we conducted experiments by removing the SRB from our proposed method, the accuracy on IC15 and SVTP noticeably decreased. This confirms the valuable role of the SRB in our text recognizer, effectively capturing differences between low and high-resolution features, aligning with our intended objective.

**Table 3:** Ablation study on several benchmark datasets.

Method	Data-Aug	SRB	SVT	SVTP	IC15	IC13
Aster	✓	×	89.5	78.5	76.1	91.8
SEED	✓	×	89.6	81.4	80.0	92.8
DAN	✓	×	89.2	80.0	74.5	93.9
Ours without SRB	✓	×	89.7	80.5	79.8	93.2
Ours	✓	✓	90.5	83.2	80.9	94.1

**Fig 8:**
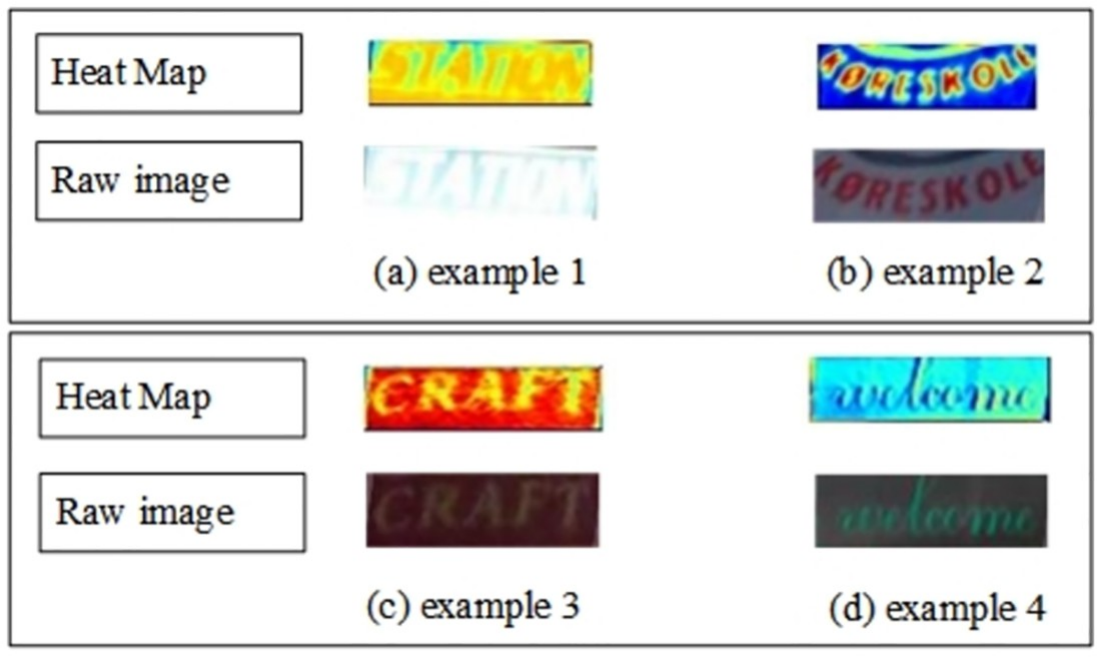
Comparisons between raw images and heat maps generated after DAM in super-resolution branch.

We also select some challenging images to evaluate the effect of the dual attention mechanism in super-resolution branch, these images are low-resolution and blurred. The feature heat maps are generated after the last DAM in super-resolution branch. As shown in Fig 8, for better comparison, we showed the heat maps in different colors, evidently, the attention features are more robust concentrated on the text area after DAM, as shown in Fig 8(b). The heat maps are clearer and have more abundant text information compared with the original images, particularly when the image is low-resolution and blurred, such as “STATION” in Example 1, Fig 8(a), which cannot be recognized by our eyes, and the “CRAFT” in Example 3, Fig 8(c), we also find that DAM is also effective in artist characters, such as “welcome” in Example 4, Fig 8(d).

#### Model analysis

We conducted an experiment to analyze model’s speed performance. For the sake of a fair comparison, we selected the NVIDIA TITAN Xp GPU as the experimental equipment, which is the same as in Wang et al. [47]. TextSR [20], which includes a GAN super-resolution branch, consumes 1.16 s per batch (128). The feature map passes through both the generator and discriminator networks resulting in slow training. As shown in Table 4, with the same GPU equipment and batch size, our proposed model consumes 0.92 s per batch (128). Furthermore, we tested the inference speed, as shown in Table 4. Our proposed model achieved the second-best performance in terms of evaluation speed. This suggests that even though we have achieved better recognition performance on multiple datasets and our speed meets the requirements for logistics scene recognition, we still need to focus on further balancing the model’s speed performance and recognition accuracy in future studies.

**Table 4:** Comparisons on model speed.

Method	Training Speed (per batch)	Evaluation speed (per image)
TextSR	1.16 s	21.83 ms
Wang et al. [46]	-	20.57 ms
Wang et al. [47]	-	18.29 ms
Ours	0.92 s	19.31 ms

**Fig 9:**
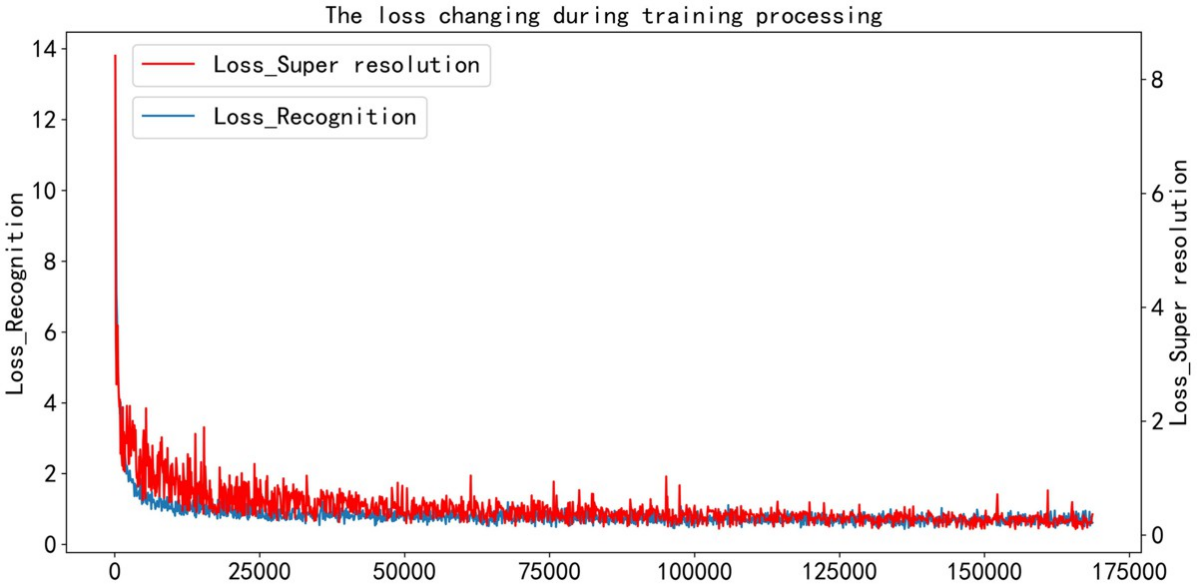
The changing of recognition loss and super-resolution loss during training.

Our model was trained for 160 thousand iterations on the SynText [38], Synth90K [39] and SynthAdd [40]. As depicted in Fig 9, the two losses converge gradually, demonstrating the crucial roles played by both the SRB and recognition module. On one hand, the SRB reduces the disparity between low-resolution and high-resolution features, and on the other hand, recognition module keeps essential information for global understanding and learns the correct sequence of characters. After 150 thousand iterations, the two curves gradually stabilized.

**Fig 10:**
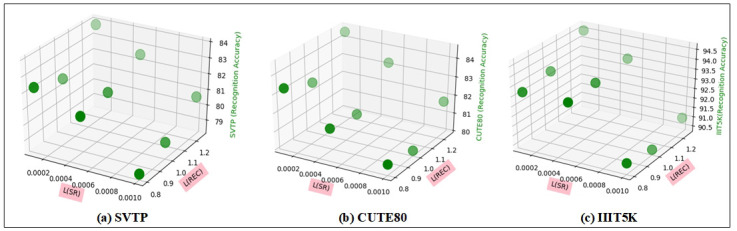
The 3D diagram displays the results of our model under different learning rates on three datasets. The X-axis represents the learning rates of SRB, the Y-axis represents the learning rates of the recognition module, and the Z-axis represents the accuracy of the dataset.

Our model comprises two primary tasks: one involves the SRB for super-resolution learning, and another is the recognition module for recognition learning. We employed distinct learning rates for these modules, the learning rate is a crucial parameter in neural networks. Throughout our experiments, we observed that a learning rate of 1 performed well for task A, whereas a learning rate of 0.0001 proved effective for task B. To delve deeper into this, we conducted multiple sets of experiments, varying learning rates from 0.001 to 0.0001 for the SRB and from 0.8 to 1.25 for the recognition module. Our evaluations encompassed challenging datasets, including SVTP, CUTE80, and IIIT5K, with results presented in Fig 10.

By observing the recognition accuracy of the SVTP, CUTE80, and IIIT5K datasets (Fig 10), we observed that the model achieved the highest performance across all three datasets when using a learning rate of 0.0001 for the SRB and 1.25 for the recognition module. Consequently, we selected these values, 0.0001 for SRB and 1.25 for the recognition module, as the optimal choices for our model.

#### Experiments on real express sheet images

Due to the rapid growth of the e-commerce industry, there has been a significant impact on the development of the logistics industry. We applied our technology to real express images to evaluate the effectiveness of our method.

**Fig 11:**
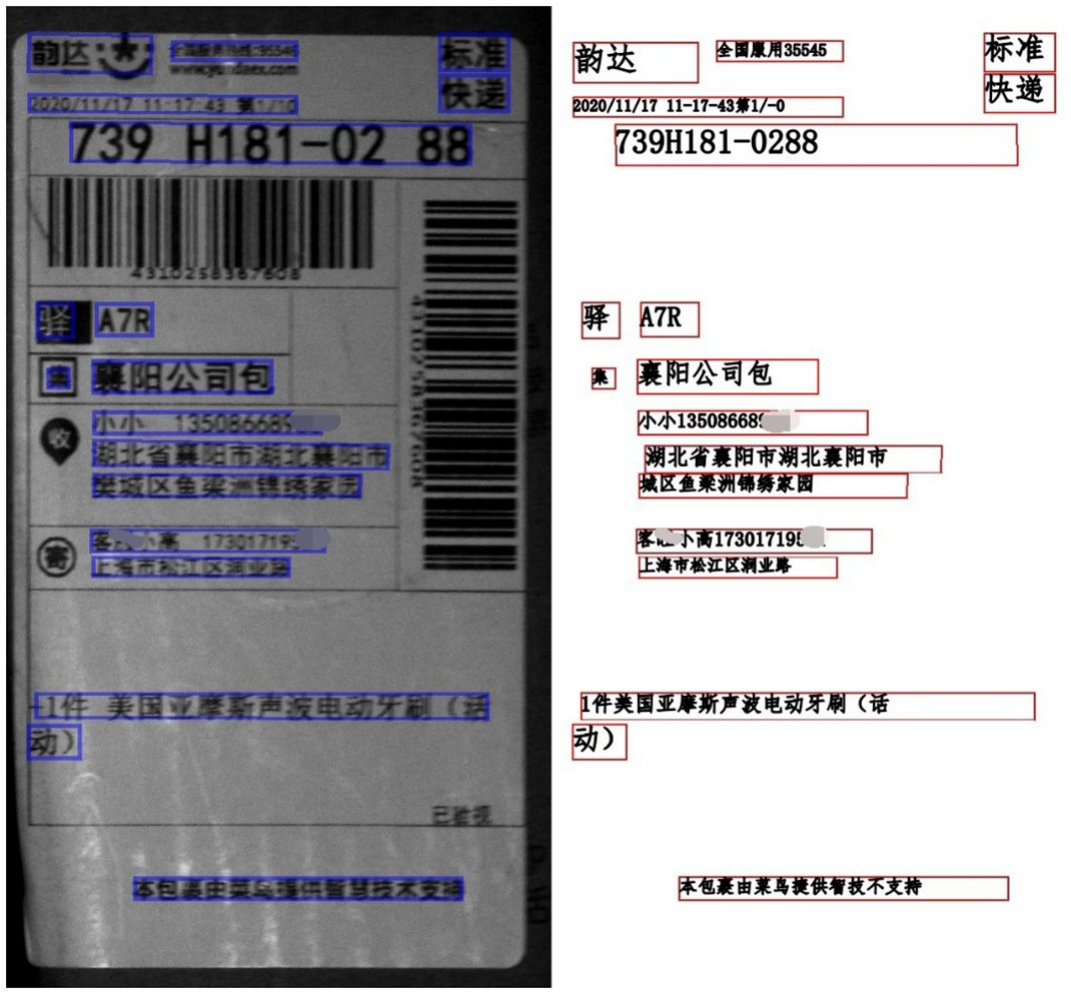
The recognition of blurred image. The area inside the blue box is the detected text region in the left side, and the right side is the recognition result corresponding to the text area one-to-one.

We start by using our text detector to identify key information on express sheet images. To safeguard customer privacy, we obscure sensitive details like names and phone numbers. In Fig 11, despite slight image blurring, our model accurately predicts vital information, including sender and recipient details (phone numbers, names, addresses), showcasing its robustness.

**Fig 12:**
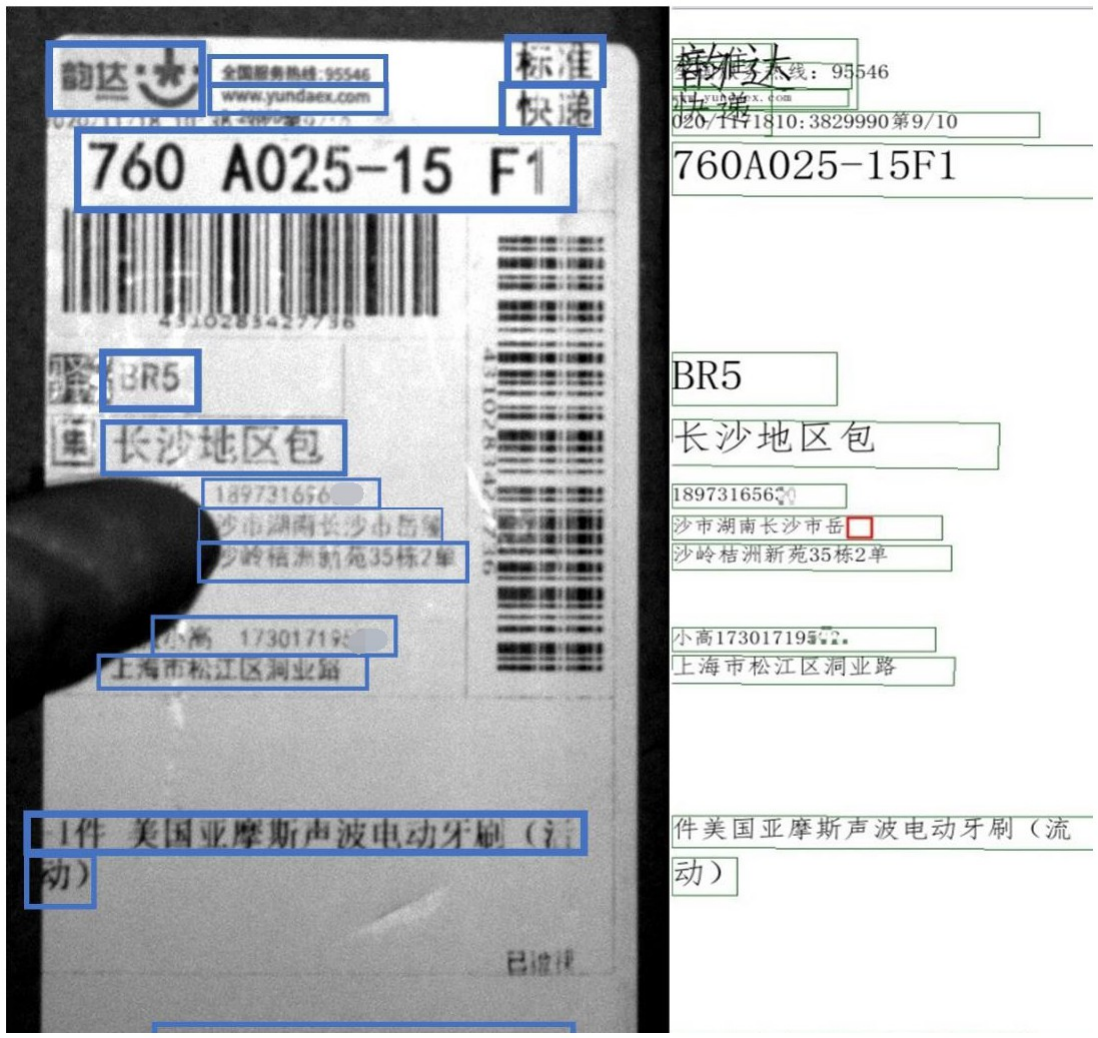
The recognition of low-resolution image. Some sensitive information was mosaicked. The left side is the detected logistics sheet image, and the right side is the recognition result corresponding recognition result for each text area.

For demonstrating our model’s ability with low-resolution express sheet images (as seen in Fig 12), we first employ the text detector to locate key regions. We then crop and input these regions into our text recognizer, as displayed in Fig 12. Despite challenges, such as low resolution and operator interference, our model accurately predicts phone numbers and Chinese addresses for both sender and recipient. Although one character is missing, as indicated in the red box in the right part of Fig 12, our model performs well with challenging characters. These results demonstrate our model’s effectiveness in handling low-resolution express sheets in the logistics industry.

#### Ablation of the hyperparameter λ

**Fig 13:**
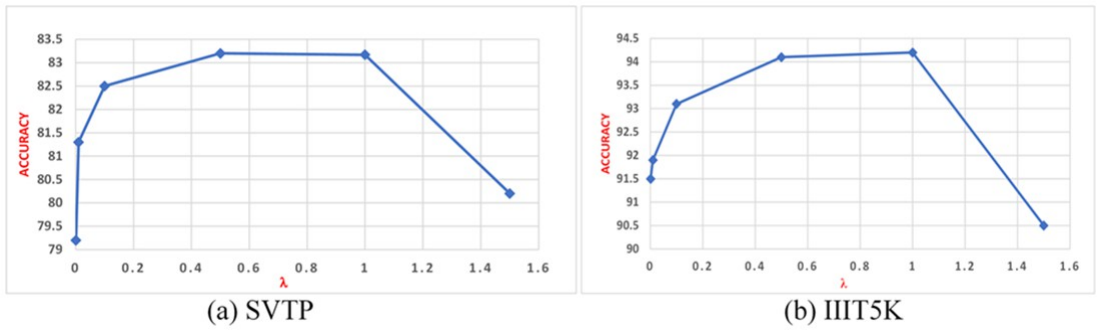
The result of our model under different value of λ. The X coordinate represents the different values of λ, Y coordinate represents the accuracy of the datasets. (a) denotes the data of SVTP, (b) denotes the data of IIIT5K.

We study the different hyperparameter of λ in Eq (17) to testify its influence on our proposed method. We set the λ from 0.001 to 1.5, and discussed the effect of different values of λ on the recognition accuracy. We selected two public datasets, SVTP and IIIT5K as the evaluation datasets. The results are shown in Fig 13, it can obviously be seen that the model reaches the best state when the value of λ reaches 0.5, when the value of λ is greater than 1, the accuracy starts to decrease. It indicates that increasing the value of λ can effectively improve text recognition performance, and the super-resolution branch indeed improves the quality of low-resolution images. However, larger λ value will make the sharing hidden layers concentrate more on low-level images, which has a negative impact on text recognition, especially when λ is greater than 1.

**Fig 14:**
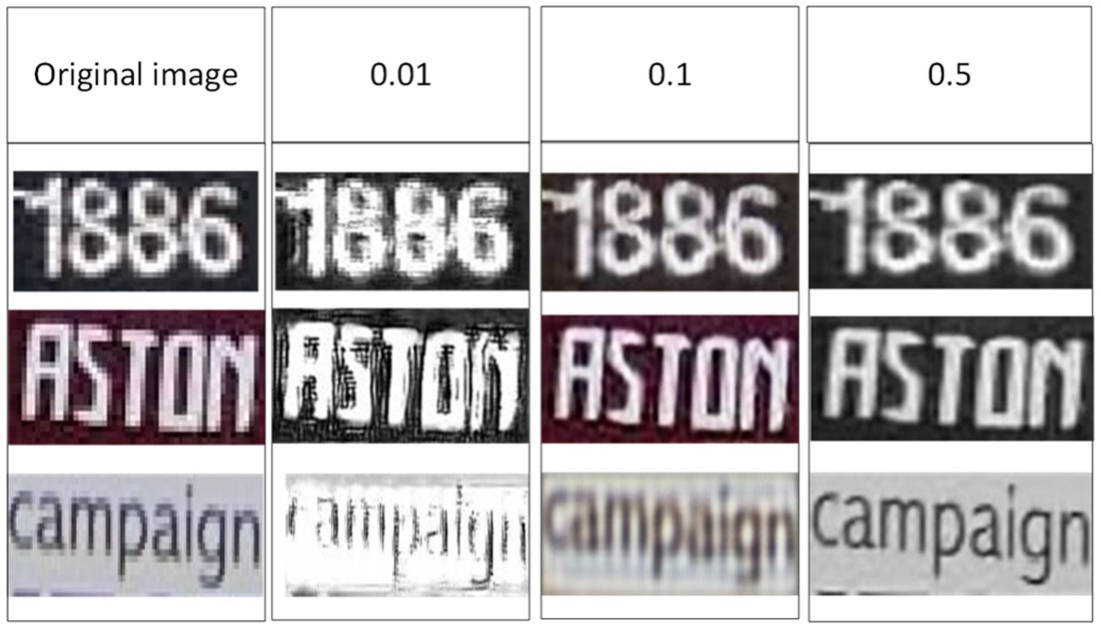
The effectiveness of SRB regarding different λ.

We also choose low-resolution and blurred images from the publicly available dataset CUTE80 to test the impact of different λ values on image enhancement. As is shown in Fig 14, it is found that when the λ equals to 0.01, it shows the bad performance. When λ equals to 0.1, it can be found that SRB can learn the features of the image, when λ equals to 0.5, it is obvious that the inferred image is clearer and higher resolution than the original image.

#### Comparisons with excellent methods

**Fig 15:**
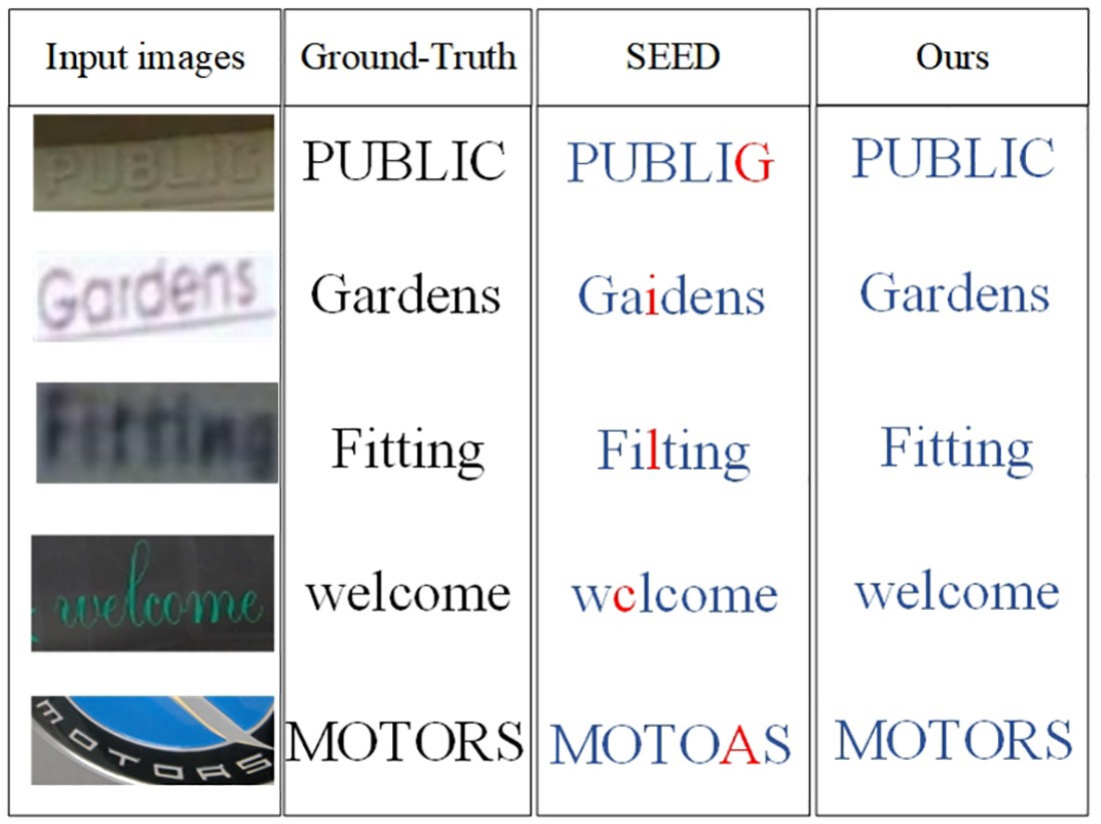
The comparison of models on low-resolution public images.

As evident from Fig 15, our model outperforms SEED [24] in recognizing challenging scenes. Despite the low-resolution and curved nature of these images, our model learns richer feature dependencies and effectively models the inter-character relationships within the text sequence. In contrast, SEED incorrectly predicted characters, as highlighted in red font.

**Fig 16:**
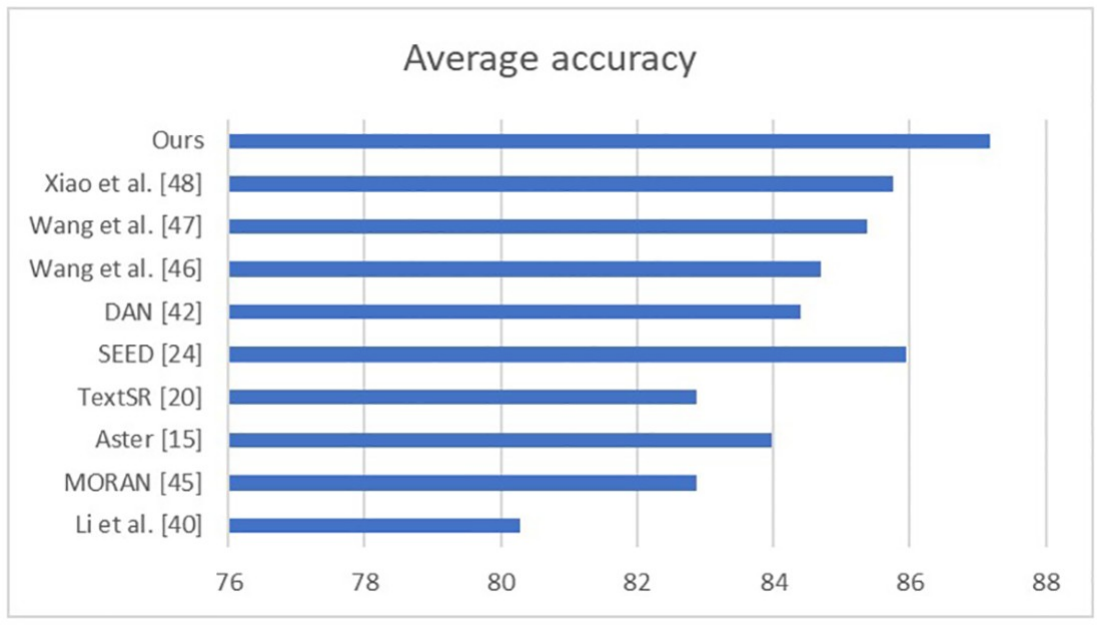
Comparison on average accuracy.

Through a comparative analysis of our model with recent outstanding methods across four datasets, namely SVT, IC13, IC15, and SVTP, our proposed model demonstrates superior performance, particularly on two low-resolution datasets (SVTP and IC15). We attribute this achievement to the pivotal role played by our proposed SRB in recognizing low-resolution images and FPN in enhancing semantic information. The visual features extracted by the CNN complement global semantic information, while the attention-based decoder learns relationships between inner characters. Fig 16 illustrates the average accuracy metric across these publicly available datasets, consistently demonstrating our model’s superior performance. Furthermore, Table 5 underscores our model’s outstanding results, particularly on SVT, IC15, and SVTP, with exceptional performance on low-resolution datasets SVTP and IC15.

**Table 5:** Comparisons with excellent models on public datasets.

Model	SVT	IC13	IC15	SVTP
Liao et al. [43]	86.4	91.5	-	-
Zhan et al. [21]	90.2	91.3	76.9	79.6
Xie et al. [44]	-	-	68.9	70.1
Li et al. [40]	84.5	91.0	69.2	76.4
MORAN [45]	88.3	92.4	74.7	76.1
Aster [15]	89.5	91.8	76.1	78.5
TextSR [20]	87.2	91.3	75.6	77.4
SEED [24]	89.6	92.8	80.0	81.4
DAN [42]	89.2	93.9	74.5	80.0
Wang et al. [47]	89.2	92.8	77.0	82.6
Xiao et al. [48]	89.7	93.6	79.1	80.6
Ours	90.5	94.1	80.9	83.2

## Conclusion

We have developed a novel text recognition model that excels on publicly available datasets, particularly when dealing with low-resolution images. Our proposed super-resolution branch effectively enhances low-resolution features, enabling us to tackle the challenges presented by low-resolution logistics images. The application of our technology holds the potential to significantly elevate the intelligence level within the logistics industry. In future research, we intend to incorporate a transformer-based decoder, as observed in recent studies, and compare its performance with the attention-based decoder employed in this study. Furthermore, we will explore the implementation of a multi-scale stage attention mechanism which was widely applied in various low-level vision tasks, with the potential to yield further performance enhancements.
